# Comparative Study on Viscoelastic Evaluation Methods of Polymer Materials Based on Ultrasonic Method

**DOI:** 10.3390/ma12182948

**Published:** 2019-09-11

**Authors:** Yuan-yuan Li, Jun-jie Chang, Lin Huang, Yong-hui Tang

**Affiliations:** 1 Key Lab of Nondestructive Testing, Ministry of Education, Nanchang Hangkong University, Nanchang 330063, China; liyy628@foxmail.com; 2 Yangtze Delta Region Institute of Tsinghua University, Jiaxing 314000, China; 3 Japan Probe, 1-1-14 Nakamura Chou Minami Ward Yokohama City, Kanagawa Prefecture, Yokohama 2320033, Japan; 4 Center for Information in Medicine, University of Electronic Science and Technology of China, Chengdu 611731, China; lhuang@uestc.edu.cn (L.H.); YonghuiTang@std.uestc.edu.cn (Y.-h.T.); 5 School of Electronic Science and Engineering, University of Electronic Science and Technology of China, Chengdu 611731, China

**Keywords:** viscoelasticity, polymer materials, spectrum analysis, attenuation coefficient

## Abstract

Rubber, as a kind of macromolecular material often used in large ships, aviation, aerospace, and other fields, has remarkable viscoelasticity at room temperature. Therefore, it is of great significance to evaluate the viscoelastic properties of polymer composites. In this paper, four kinds of rubber materials are taken as research objects. Based on the principle of ultrasonic detection, the viscoelastic evaluation of the sample materials is carried out through experiments and simulations. On the basis of previous research, the surface reflection method (SRM) and the bottom reflection method (BRM) are compared in depth. First, the spectrum of received signals is analyzed, and the storage elastic modulus, loss elastic modulus, attenuation coefficient and loss tangent value are obtained. Secondly, the results of the BRM and the SRM are compared and analyzed in the frequency domain of –6 dB. The results show that both the SRM and BRM are feasible in the evaluation of the viscoelasticity of the material, and the variation trends observed for the above-mentioned parameters in the effective frequency domain are consistent, especially at the center frequency. Finally, aiming at the mode transformation of the acoustic wave around the ultrasonic sensor, the practical performance of the surface reflection method is optimized by increasing the diameter of the ultrasonic sensor.

## 1. Introduction

Ultrasonic technology is widely used in various applications as a method for the non-destructive testing of materials and structures. The detected medium can judge the constituent materials, manufacturing conditions, defects, and deterioration states based on the information attached to the reflected waveform [1,2]. In the case of composite materials, due to various micromechanical theories, the elastic properties and the material properties of the constituent phases (base metal and strengthening phase) are related to the existing state of internal defects [3,4,5]. Through the study of ultrasonic propagation characteristics and micromechanical modeling, a large amount of microstructure information of the composite material can be obtained from the reflected waveform. In the microstructural evaluation of composite materials, the attenuation coefficient of the material and the loss tangent value are important parameters for the viscoelastic evaluation of materials. Regarding the ultrasonic attenuation characteristics of materials, from the perspective of scattering theory, there is also a great correlation between the grain size and the attenuation coefficient, which can be explained by scattering theory [6,7,8,9]. Some scholars have tried to extract microscopic information through the measurement of the attenuation coefficient of composite materials, such as the study of the attenuation characteristics of polymer matrix composites by scattering theory, or the correlation between fatigue damage and attenuation changes [10].

Rubber materials have excellent resistance to high- and low-temperature, aging, electrical insulation, physiological inertness, etc., and are often used in large ships, aviation, and aerospace [11,12]. The solid mechanical properties of rubber materials are not only elastic, but also viscoelastic at room temperature. In rubber objects, ultrasonic waves propagate at an inherent velocity in the material and are attenuated by viscoelasticity. Therefore, by measuring the velocity and attenuation coefficient of ultrasonic propagation in the material, the viscoelasticity (complex modulus) of the material can be evaluated [13]. The propagation speed of ultrasonic waves in rubber materials is closely related to the viscoelasticity of rubber materials. Therefore, a decrease in the elastic modulus caused by a defect or damage can be evaluated by the propagation speed. Also, since the ultrasonic attenuation characteristic in the rubber material is affected by scattering caused by coarse grains or defects, in addition to the influence of viscoelasticity, internal defects or damage are reflected as changes in the attenuation characteristics. As the ultrasonic characteristics in polymer materials, the phase velocity demonstrates dispersion characteristics and the attenuation coefficient is proportional to the frequency. On the other hand, according to Boltzmann’s viscoelastic linear-response theory, the two can be represented by the real and imaginary parts of a complex number. By making it correspond to the Kramers–Kronig dispersion relation, a general equation that relates the attenuation coefficient to the phase velocity can be obtained, and the O’Donnell equation is derived by appropriate approximation [14].

The method of deriving the attenuation coefficient using wave theory has been studied extensively, and the method of using the retardation material and the first bottom reflection of the sample by Hirao shows the consistency between the above theory and the experimental values [14]. Ivey performs a tensile test under low-frequency conditions to obtain Young’s modulus and calculate a value of 100 kHz or more from the measured data of the ultrasonic longitudinal wave, and the result can be derived from continuous tanδ [15,16]. Gerspacher et al. used the attenuation coefficient of the ultrasonic wave as the evaluation method of the tire traction test. In this study, evaluation of the high-frequency viscoelasticity of the carbon black filler content is performed, the frequency at which the attenuation coefficient reaches its maximum value is determined, and the temperature dependence of the attenuation coefficient is studied quantitatively. It can be seen that ultrasonic radiation is effective for evaluating the high-frequency dynamic properties of rubber materials [13,17]. Due to the large attenuation of the rubber material, it is difficult to obtain high-precision resolution ultrasonic signals [18]. Ultrasonic research on rubber materials is more scarce than that for other materials, but in recent years, with the development of high-power ultrasonic transmitters, amplification and filtering, signal processing and other technologies, it is possible to solve the noise phenomena that are difficult to overcome in traditional measurements to some extent. 

Previously, in the study of the evaluation of the viscoelasticity of rubber materials by ultrasonic waves, the consistency of the viscoelasticity evaluation results of the same material by the ultrasonic bottom reflection method and the traditional DMA method has been proved by experiments and simulations, the BRM measured loss tangent (0.0478) are within 3.4% of results by DMA (0.0462) that were measured in [19], it also proves that the surface reflection method is feasible. However, the research on the attenuation coefficient in the bottom reflection method process is not deep enough, and the surface reflection method and the bottom reflection method are not studied in the frequency domain [19,20,21,22]. Based on the above, this paper uses practical rubber materials, the ultrasonic detection system performs spectral analysis on the obtained output signal, the characteristics of the rubber materials are evaluated by analyzing the relationship between the attenuation coefficient, the storage modulus, the loss modulus, the loss tangent and the frequency. At the same time, using the PZFlex analysis method, the same analytical model as the experimental measurement was studied and the ultrasonic propagation speed in the rubber material was clarified; the SRM and the BRM experimental and analysis results were compared and ways to optimize experimental results were explored 

## 2. Ultrasonic-based Viscoelastic Evaluation Method

### 2.1. Viscoelastic Evaluation Method

**Figure 1:**
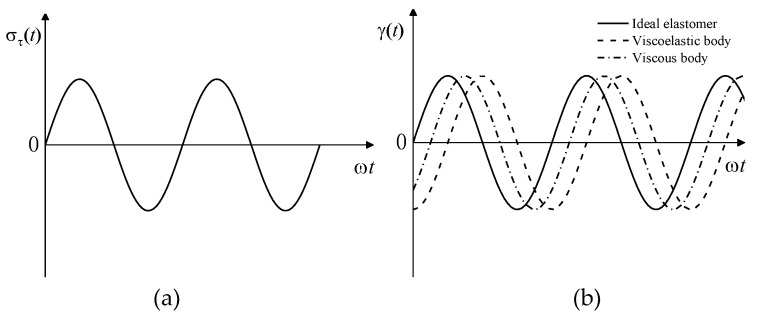
Strain response of materials to a sinusoidal alternating stress: (**a**) sinusoidal alternating stress; (**b**) strain response.

There are two types of studies on the viscoelastic properties of materials: one is static viscoelasticity research and the other is dynamic viscoelasticity research [23,24,25]. In the actual use of materials, the force is usually dynamic; therefore, dynamic viscoelasticity can better reflect the performance of the material under actual use conditions. In dynamic mechanical experiments, the most common sinusoidal stress in Figure 1a is applied to the object. Stress expression, using dynamic shear as an example, is as follows:

(1) $$\sigma_{\tau }\left(t\right)=\sigma_{\tau 0}\sin\omega t$$

where $\sigma_{\tau 0}$ is the stress amplitude and ω is the angular frequency.

(1) For an ideal elastomer, the response of the strain to the stress is instantaneous, so the strain response is a sinusoidal function in phase with the stress, as shown by the solid line in Figure 1b. The strain can be expressed as:

(2) $$\gamma \left(t\right)=\gamma_{0}\sin\omega t$$

where $\gamma_{0}$ is the strain amplitude.

(2) For an ideal viscosity, the strain can be expressed as:

(3) $$\gamma \left(t\right)=\gamma_{0}\sin\;(\omega t-\pi /2)$$

The strain lags behind the stress by π/2, as shown by the dashed line in Figure 1b.

(3) For viscoelastic materials, the strain lags behind the stress by a phase angle δ (0< δ < π/2), as shown by the dotted line in Figure 1b, that is, the stress is earlier than the strain by a phase angle. when the strain is given as:

(4) $$\varepsilon_{\tau }\left(t\right)=\varepsilon_{0}\sin\omega t$$

the stress can be described as:

(5) $$\sigma_{\tau }\left(t\right)=\sigma_{\tau 0}\sin\;(\omega t+\delta )$$

Expansion of the above equation can be expressed by the following equation:

(6) $$\sigma \left(t\right)=\sigma_{0}\sin\omega \mathrm{tcos}\delta +\sigma_{0}\cos\omega \mathrm{tsin}\delta$$

That is the sum of $\sigma_{0}\sin\omega \mathrm{tcos}\delta$ (the stress in phase with strain, the main power of elastic deformation) with $\sigma_{0}\cos\omega t\sin\delta$(the stress that is π/2 out of phase with strain, and it is deformed by the viscosity of the deformation, which is consumed when the frictional resistance is overcome).

The main parameters of dynamic viscoelasticity are the storage modulus $G{}^{\prime }$, loss modulus $G{}^{\prime \prime }$ and loss tangent value tanδ [24,25]. The storage modulus $G{}^{\prime }$ can be defined as the stress-to-strain amplitude ratio in the same phase; loss modulus $G{}^{\prime \prime }$ can be defined as the stress-to-strain amplitude ratio with a difference of π/2:

(7) $$G{}^{\prime }=\frac{\sigma_{0}\cos\delta }{\varepsilon_{0}}=\frac{\sigma_{\infty }}{\varepsilon_{0}}\cos\delta$$

(8) $$G{}^{\prime \prime }=\frac{\sigma_{0}\sin\delta }{\varepsilon_{0}}=\frac{\sigma_{0}}{\varepsilon_{0}}\sin\delta \;$$

(9) $$\sigma \left(t\right)={G{}^{\prime }\varepsilon }_{0}\sin\omega t+{G{}^{\prime \prime }\varepsilon }_{0}\cos\omega t$$

Therefore, using $G^{*}$ as the complex modulus, it can be obtained from the mathematical complex form:

(10) $$G^{*}=G^{{}^{\prime }}+iG^{{}^{\prime \prime }}$$

The loss tangent value is calculated as:

(11) $$\tan\delta =\frac{G{}^{\prime \prime }}{G{}^{\prime }}$$

Therefore, the loss angle δ can be used to evaluate the viscoelastic properties of the material.

### 2.2. Bottom Reflection Method to Evaluate Material Viscoelasticity

**Figure 2:**
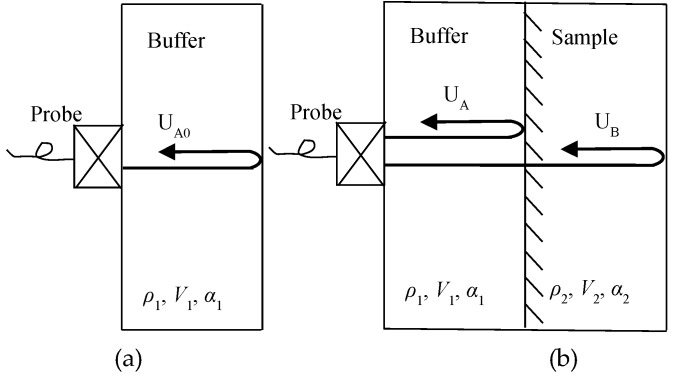
Schematic diagram of ultrasonic propagation of the bottom reflection method (BRM): (**a**) With no sample: reflected wave A_0_ of buffer and the air interface; (**b**) with a sample: reflected wave A of buffer and the sample interface, reflected wave B of sample and the air interface.

In the ultrasonic direct-contact method, the following method is used to correct the reflection and transmission characteristics at the transducer and sample boundaries. As shown in Figure 2a, the buffer material density is $\rho_{1}$, the longitudinal wave propagation velocity is $v_{1}$, and the attenuation is $\alpha_{1}$. The transducer is placed directly on the buffer material and the acoustic coupling between the transducer and the retardation material is achieved with high precision. The transducer emits ultrasonic waves, the waves enter the buffer material and are reflected by the bottom of the buffer material, then received by the transducer. The transducer is acoustically coupled to the sample by the buffer. As shown in Figure 2b, the density of the sample is represented by $\rho_{2}$, the longitudinal wave propagation velocity is $v_{2}$, and the attenuation is $\alpha_{2}$. At this setting, the ultrasonic wave is incident. The pulse reaches the bottom of the buffer, a part of the ultrasonic wave is reflected by the surface of buffer and the sample, and another part of the ultrasonic wave enters the sample and is reflected by the bottom of the sample.

In Figure 2a, U_A0_ is the reflected echo from the air interface through the buffer material. In Figure 2b, U_A_ is the reflected echo from the sample interface and U_B_ is the ultrasonic wave that travels through the sample. Spectrum analysis was performed on these reflected echoes by Fourier transform [26,27,28]. When the absolute values of the three echoes in the frequency domain are set to A_0_(*f*), A(*f*), and B(*f*), the attenuation coefficient of the sample cab be formulated as [29,30,31]:

(12) $$\alpha \left(f\right)=\frac{1}{2h}\ln\left(\frac{A_{0}{\left(f\right)}^{2}-A{\left(f\right)}^{2}}{A_{0}\left(f\right)B\left(f\right)}\right)$$

where *h* is the thickness of the sample and *α* is the attenuation coefficient (1/cm) of the sample. The phase spectra of A(*f*) and B(*f*) can be obtained from the real part and the imaginary part of A(*f*) and B(*f*) in the frequency domain analysis. Then, the phase velocity V_p_(*f*) of the ultrasonic propagation of the sample material can be described as:

(13) Vp(f)=2hωtan−1Im[B(f)]Re[B(f)]−tan−1Im[A(f)]Re[A(f)]+2Nπ+ωT

where *f* is the frequency, ω is the angular frequency (ω=2π*f*), and T is the start time difference of the fast Fourier transform (FFT) time window of A(*f*), B(*f*). Since the arbitrariness of 2Nπ is generated in the phase angle based on the arctangent, phase correction is performed. In addition, according to the complex elasticity theory, the storage modulus $G{}^{\prime }$, the loss elastic modulus $G{}^{\prime \prime }$ and specific loss tangent tanδ of the longitudinal ultrasonic wave in the medium are derived as follows, where *ρ* is the density, $\frac{\alpha V_{p}}{\omega }\ll 1$.

(14) $$G^{{}^{\prime }}=\rho V_{p}{}^{2},\;G{}^{\prime \prime }=\frac{2\alpha \rho V_{p}{}^{3}}{\omega }=\frac{2{\alpha V}_{p}}{\omega }G{}^{\prime }$$

(15) $$\tan\delta =\frac{G{}^{\prime \prime }}{G{}^{\prime }}=\frac{2{\alpha V}_{p}}{\omega }$$

The higher the frequency of the ultrasonic wave that reaches the sample material, the greater the attenuation caused by the buffer material, and the more obvious the difference in the frequency domain analysis is. Therefore, the frequency characteristics of the attenuation coefficient, phase velocity, and loss tangent value tanδ are studied in the frequency range of –6 dB with respect to the amplitude spectral density $A_{0}\left(f\right)$ of the reflected wave on the bottom of the retardation material.

The above describes the measurement method of the viscoelasticity of the sample material by the surface reflection waveform and the bottom reflection waveform of the sample, that is, the bottom reflection method (BRM) [17,20]. However, in actual cases, the shape of the bottom of the material and the structure are different, and it is not limited to the plane. At this time, the correct reflection waveform cannot be measured from the bottom of the sample.

### 2.3. Evaluation of Viscoelastic Properties of Materials by the Surface Reflection Method

For non-planar bottom materials, BRM cannot measure the sample viscoelasticity. Therefore, a surface reflection method (SRM) is used. The complex acoustic impedance is obtained by delaying the reflected wave of the material at the air interface when there is no sample and the reflected wave of the buffer material at the sample interface when there is a sample. 

The reflection coefficient of the interface between the buffer material and the sample can be obtained from the following formulation:

(16) $$R_{2}=\frac{Z_{2}-Z_{1}}{Z_{2}+Z_{1}}$$

Among them: the acoustic impedance of the buffer material is $Z_{1}$, the acoustic impedance of the sample is $Z_{2}$, the reflection coefficient of the interface between the buffer material and the sample is *R_2_*. At this time, if the complex amplitude spectrum of the Fourier transform based on U_A0_ and U_A_ is set to A_0_ and A, $A^{*}$ is the incident wave emitted to the buffer material when there is no sample, and the reflection from the air layer is the total reflection. Then the reflection coefficient $R_{1}$ of the buffer material to the air can be expressed as:

(17) $$R_{1}=\frac{A_{0}}{A^{*}}=-1,\;A^{*}=-A_{0},\;R_{2}=\frac{A}{A^{*}}=-\frac{A}{A_{0}}$$

where $A^{*}$ is assumed to be the amplitude of the incident wave from the buffer material to the air. $A_{0}=\left|A_{0}\right|e^{{i\theta }_{A0}},\;A=\left|A\right|e^{{i\theta }_{A}}$, ${\;A}_{0}$ is the reference reflected wave of the buffer material, and A is the surface reflected wave of the sample. Therefore, the reflection coefficient $R_{2}$ between the retardation material and the sample is as follows:

(18) $$R_{2}=-\frac{\left|A\right|e^{i\theta_{A}}}{\left|A_{0}\right|e^{i\theta_{A0}}}=-\left|R_{2}\right|e^{-i\theta }$$

where $\left|R_{2}\right|=\left|A\right|/\left|A_{0}\right|,\;\theta =\theta_{A0}-\theta_{A}$. The amplitude spectrum is obtained by Fourier transform of the reflected wave to obtain |A|*,* $\left|A_{0}\right|$. $\theta_{A_{0}}$*,* $\theta_{A}$ is the amplitude spectrum obtained by its arctangent. However, since the phase angle produces arbitrariness of 2Nπ, it is corrected to a smooth curved shape by adding 2Nπ. By combining Equation (16) and Equation (18), the acoustic impedance of the sample can be expressed as:

(19) $$Z_{2}=\frac{1-\left|R_{2}\right|e^{-i\theta }}{1+\left|R_{2}\right|e^{-i\theta }}Z_{1}=\frac{1-{\left|R_{2}\right|}^{2}+2i\left|R_{2}\right|\sin\theta }{1+{\left|R_{2}\right|}^{2}+2\left|R_{2}\right|\cos\theta }Z_{1}$$

The real part is *X*, the imaginary part is *Y*:

(20) $$X=\frac{1-{\left|R_{2}\right|}^{2}}{1+2\left|R_{2}\right|\cos\theta +{\left|R_{2}\right|}^{2}}Z_{1},\;Y=\frac{2\left|R_{2}\right|\sin\theta }{1+2\left|R_{2}\right|\cos\theta +{\left|R_{2}\right|}^{2}}Z_{1}$$

When the longitudinal wave is incident:

(21) $$G^{*}=G{}^{\prime }+iG{}^{\prime \prime }=\frac{Z_{2}{}^{2}}{\rho_{2}}$$

where $G^{*}$ is the tensile composite elastic modulus.

(22) $$G^{{}^{\prime }}=\frac{X^{2}-Y^{2}}{\rho_{2}}=\frac{Z_{1}{}^{2}}{\rho_{2}}\frac{{\left(1-{\left|R_{2}\right|}^{2}\right)}^{2}-4{\left|R_{2}\right|}^{2}\sin^{2}\theta }{{\left(1+2\left|R_{2}\right|\cos\theta +{\left|R_{2}\right|}^{2}\right)}^{2}}\;G{}^{\prime \prime }=\frac{2XY}{\rho_{2}}=\frac{Z_{1}{}^{2}}{\rho_{2}}\frac{4\left|R_{2}\right|\left(1-{\left|R_{2}\right|}^{2}\right)\sin\theta }{{\left(1+2\left|R_{2}\right|\cos\theta +{\left|R_{2}\right|}^{2}\right)}^{2}}\;\tan\delta =\frac{G{}^{\prime \prime }}{G{}^{\prime }}=\frac{4\left|R_{2}\right|\left(1-{\left|R_{2}\right|}^{2}\right)\sin\theta }{{\left(1-{\left|R_{2}\right|}^{2}\right)}^{2}-4{\left|R_{2}\right|}^{2}\sin^{2}\theta }$$

The above describes the evaluation method of dynamic viscoelasticity of materials based on ultrasonic waves.

## 3. Viscoelastic Evaluation of Different Rubber Materials by the BRM

### 3.1. Simulation Analysis Feasibility Verification

**Figure 3:**
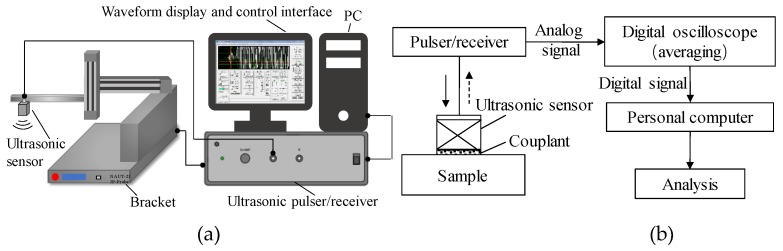
Experimental system and principle diagram. (**a**) Ultrasonic pulser/receiver and fixed fixture used in actual measurements; (**b**) the measurement system of the direct contact method for the ultrasonic reflection experiment.

In this paper, the relationship between attenuation characteristics, viscoelasticity of different rubber materials and frequency is studied by both simulation and experiment. PZFlex software (PZFlex, LLC, Cupertino, USA) was developed for ultrasound detection and has been widely used in various engineering fields in recent years, which can simulate the process of wave propagation and obtain the ultrasonic receiving time domain signal. In this software, a number of advanced theories, algorithms, and parallel techniques are used to solve models of tens of millions of units. PZFlex is based on the finite element with an explicit-implicit solver and uses the latest parallel computing technology. Compared with other finite element software, users can realize the calculation of very large-scale models on an ordinary PC. In this study, the software can not only verify the accuracy of the experimental results, but also can change different parameters easily and then calculate the results quickly after the model is established, which is economical and efficient. A JAPAN 600C (Japan Probe Co. Ltd, Yokohama, Japan) ultrasonic pulser/receiver, fixed fixture (IAI Co. Ltd, Shizuoka, Japan) was used for the measurements, as shown in Figure 3a. The measurement system shown in Figure 3b used the direct contact method for the ultrasonic reflection experiment. The ultrasonic sensor was placed on the buffer, and the coupling agent was used to achieve good coupling. The probe simultaneously transmits and receives ultrasonic waves, which was converted into a digital signal through a digital oscilloscope and transmitted to the PC terminal for analysis and waveform display. The details of measurement conditions are shown in Table 1.

Firstly, a comparison was conducted on whether the attenuation characteristics of buffer materials could be ignored in the simulation process. The size of the model was the same as that of the samples used in the experiment. The buffer material thickness was expressed by *L_1_* and the sample thickness by *L_2_*. The schematic of the experiment is shown in Figure 2b. Acrylic resin blocks ($\;\rho_{1}$ = 1187 kg/m^3^, $\nu_{1}$ = 2670 m/s, *L_1_* = 35mm) are used as the buffer material and the sample material is iron ($\rho_{2}$ = 7700 kg/m^3^, $\nu_{2}$ = 5850 m/s, *L_2_* = 20mm). The ultrasonic contact probe was manufactured by Japan Probe Co. Ltd (Yokohama, Japan) with an excitation frequency of 1 MHz.

**Figure 4:**
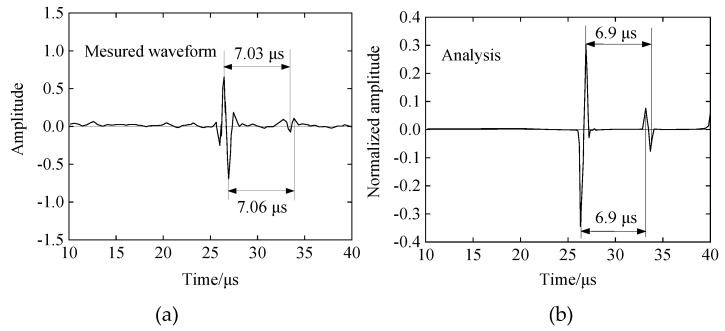
Experimental and analytical comparison of reflected waveforms demonstrates that the attenuation of the buffer material can be ignored in the simulation. (**a**) Experimentally measured reflected waveform (actual); (**b**) analyzed reflected waveform with PZFlex (theoretical).

The reflection waveform obtained by experimental measurement and the reflection waveform obtained by ultrasonic propagation analysis are respectively shown in Figure 4a,b. When comparing time-domain waveforms, because the measured value uses the value converted to the electrical signal (Volt), the scale interval of the horizontal axis of the two figures is unified to 5 μs. The waveform diagram only shows the range it takes to receiving the reflected waveform. The shape of the wave packets obtained in the comparative experimental measurement and the simulation analysis are almost the same, and the reflected waves are received at the interfaces of the buffer-material–iron interface and the iron–air layer, and the phases are substantially reversed. According to the speed of sound argument and the model size, theoretical analysis and the calculation of two reflection wave times of 6.9 μs can be calculated while the measurement of the reflection waveform peak interval is 7.03~7.06 μs. Analysis error reason: on the one hand, the experimental incident wave is a square pulse wave, but the incident wave analyzed is a sine wave; on the other hand, during the test, the actual attenuation coefficient of the buffer material is not 0, but the attenuation coefficient is set to zero in the analysis. However, the error between the analytical result and the experimental measurement is only about 2%. Therefore, by the above comparative analysis, the attenuation of the buffer material can be ignored in the simulation process.

### 3.2. Research on the BRM through Experimentation and Simulation

The four rubber materials used for the evaluation of viscoelasticity were made of silicon (Si) added to base rubbers, and were adjusted to 40-degree hardness type A rubber according to JIS K6253, respectively [32,33,34,35,36], the three basic materials are ethylene propylene diene monomer (EPDM), styrene butadiene rubber (SBR), butadiene rubber (BR). Among them, EPDM is a copolymer of ethylene, propylene and a small amount of non-conjugated diene. It is widely used in the industry because its main chain is composed of chemically stable saturated hydrocarbons and contains only unsaturated double bonds in the side chain. It is widely used in automotive rubber parts and building construction rubber products due to its heat resistance, weather resistance and aging resistance. SBR is a general-purpose synthetic rubber made by copolymerization of butadiene and styrene. Due to its excellent properties such as elasticity, strength properties, wear resistance, good processability and relatively low price, it is often used as general industrial products such as hoses, conveyor belts and footwear materials. BR has better cold resistance, aging resistance and wear resistance, and has high elasticity, small dynamic heat, miscibility, high filling, moldability, mold fluidity, and processability. However, the cutting performance is poor, so it is often used for blends of SBR or natural rubber (NR) rather than alone. The samples to be evaluated are named as: EPX-46, OR4Si, ORHS4Si, B4Si, which EPX-46 belongs to the EPDM material, OR4Si belongs to SBR1 material, ORHS4Si belongs to SBR2 material, and B4Si belongs to BR material. They are all named according to the hardness of the material, the physical properties of the filling material, etc. The sound velocity of rubber materials is determined by the time difference between the peaks of the secondary reflected waves and the thickness of the four kinds of samples [37]. The physical property values of these materials to be evaluated are shown in Table 2.

**Figure 5:**
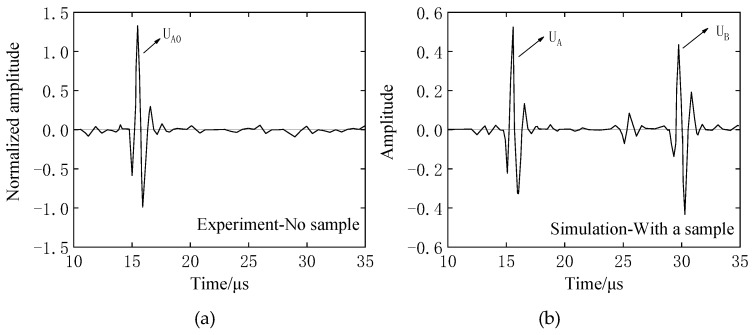
Evaluation of the viscoelastic experimental waveform of rubber materials by the BRM: (**a**) with no sample—receive waveform U_A_ with acrylic resin buffer; (**b**) with a sample—the valid signals are U_A_ and U_B_ with EPX-46 material as an example.

The buffer material is acrylic resin ($\rho_{1}$ = 1187 kg/m^3^, $\nu_{1}$ = 2670 m/s, *L_1_* = 20 mm), and the sample is the above four different rubber materials, *L_2_* = 10 mm. During the test, sensor–buffer-material and buffer–block-sample coupling are well coupled as two separate couplings, the sensor diameter is *d* = 10mm, and the excitation frequency is *f* = 1 MHz. The receiving waveform obtained by the measurements of four rubber materials, but show only EPX-46 as an example. In the case of only the buffer, the receiving waveform is as shown in Figure 5a. After the sample is added at the end of the buffer, the receiving waveform is as shown in Figure 5b. 

**Figure 6:**
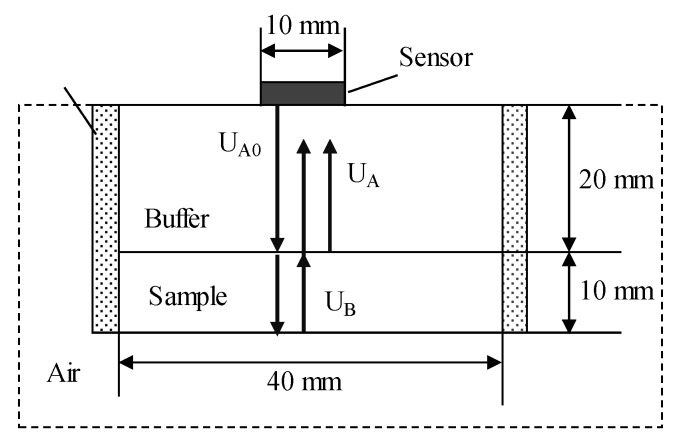
Schematic diagram of the simulation analysis model using PZFlex software. The size settings of the probe, buffer, and sample are consistent with the experiment.

**Figure 7:**
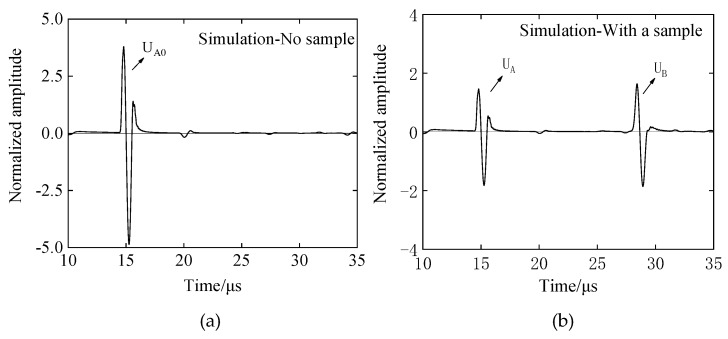
Evaluation of the viscoelastic simulation waveform of rubber materials by the BRM: (**a**) with no sample—receive waveform UA with acrylic resin buffer; (**b**) With a sample—the valid signals are U_A_ and U_B_ with EPX-46 material as an example.

The analysis model and experimental conditions were set to be the same. The simulation analysis model is shown in Figure 6. The sample and the buffer are in the air medium, the left and right boundary conditions are the absorption boundary, the upper and lower boundaries are the roller support, and the ultrasonic wavelength is represented by λ. Then, the model element is divided into λ/80. Also taking EPX-46 material as an example, the receiving waveform is shown in Figure 7.

**Figure 8:**
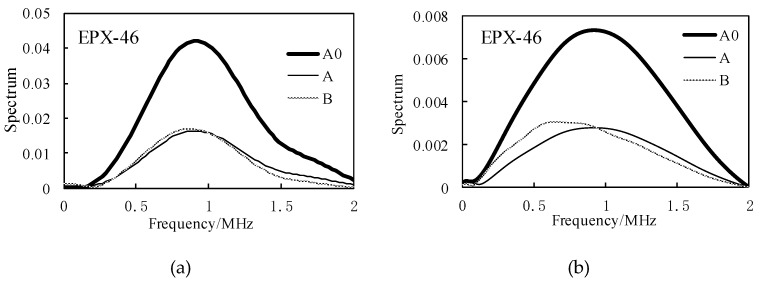
Spectrum analysis of received reflected waves in the presence of samples, taking EPX-46 as an example: (**a**) received waveform by experiment; (**b**) received waveform by simulation.

As shown in Figure 6 and Figure 7, when there is only a buffer, the reflected wave on the bottom of the buffer is received at 14.98 μs. At this time, the difference between the buffer and the air medium is large, and most of the ultrasonic energy is reflected on the bottom of the buffer and received by the sensor, so the amplitude of the reflected echo is higher. When the buffer is well coupled with the sample to be tested, part of the ultrasonic wave is reflected by the bottom surface of the buffer and received at 14.98 μs. The remaining ultrasonic waves are transmitted into the sample, and then reflected by the bottom surface of the sample before being received by the receiving sensor at 28.65 μs. Therefore, U_A_ < U_A0_. The amplitude spectrum after FFT of the reflected wave obtained from the rubber material of EPX-46 is shown in Figure 8.

**Figure 9:**
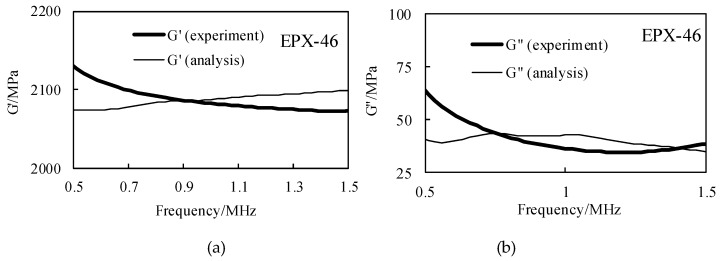
The storage modulus and the loss modulus are calculated according to the received signal by the BRM, and the experimental results are compared with the analytical results in the effective frequency domain range, taking EPX-46 as an example: (**a**) the results of $G^{{}^{\prime }}$; (**b**) the results of $G^{{}^{\prime \prime }}$.

The spectral analysis and amplitude distribution characteristics of the above 0–2 MHz range are characterized as follow: the center frequency is about 0.9 MHz, and the amplitude decreases when the frequency is gradually increased or decreased. When only the buffer material is used, the corresponding A_0_(*f*) spectrum value is higher; in the case of samples, the values of A(*f*) and B(*f*) are smaller, about 0.5 times those of A_0_(*f*). According to Equations (12)–(14), the storage modulus $G^{{}^{\prime }}$ and the loss modulus $G^{{}^{\prime \prime }}$ can be obtained, and the results are shown in Figure 9.

**Figure 10:**
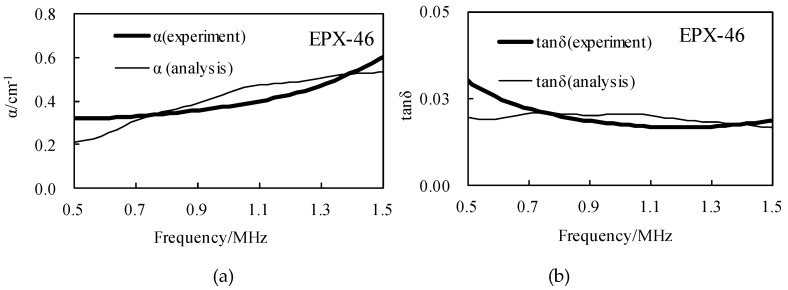
The attenuation coefficient and the loss tangent are calculated by the BRM, and the experimental results are compared with the analytical results in the effective frequency domain range, taking EPX-46 as an example: (**a**)the results of *α*; (**b**) the results of tanδ.

Further, the relationship between the attenuation coefficient α, the loss tangent value tanδ and the frequency can be obtained according to Equations (12)–(15), and the result of the frequency of the effective band of 0.5 MHz to 1.5 MHz is as shown in Figure 10. In Figure 9, the storage modulus $G^{{}^{\prime }}$ obtained by experiment and simulation varies from 2075 MPa to 2126 MPa, and the experimental results are gradually reduced in the frequency domain, the simulation results are gradually increased in the frequency domain, but they are similar at the center frequency of 1MHz; the loss modulus $G^{{}^{\prime \prime }}$ varies from 33 MPa to 63 MPa, the experimental and simulation results show a decreasing trend in the frequency domain. In Figure 10, the attenuation coefficient α increases with the increase of frequency, which is consistent with the characteristics of ultrasonic propagation. The loss tangent tanδ decreases with the increase of frequency, and for these two parameters, their experimental results and simulation results are similar under the same conditions, which proves the accuracy of the BRM results. The attenuation coefficient α and the loss tangent value tanδ of the four rubber materials at the center frequency are shown in Table 3 and Table 4, respectively.

From the above results, it is known that the attenuation coefficient obtained by experimental measurement and numerical analysis of BRM shows a linear increase trend with respect to frequency variation. According to the evaluation results of all rubber materials, the experimental measurement results are consistent with the analysis results. In the experimental measurement, the results vary more rapidly with the change in the frequency. In the frequency range below 1.2 MHz, the two methods of experimental measurement and numerical analysis are more coincident.

## 4. Viscoelastic Evaluation by the SRM and Comparative Analysis of Results

### 4.1. Research on the SRM through Experimentation and Simulation

**Figure 11:**
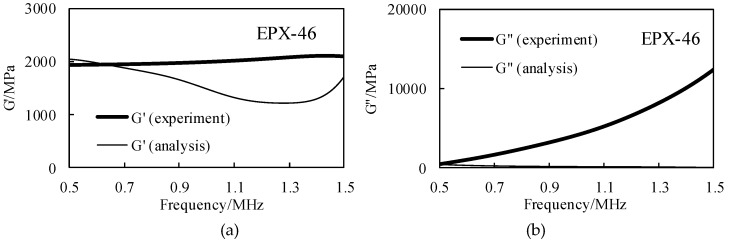
The storage modulus and the loss modulus are calculated by the surface reflection method (SRM), and the experimental results are compared with the analytical results in the effective frequency domain range, taking EPX-46 as an example: (**a**) the results of $G^{{}^{\prime }}$; (**b**) the results of $G^{{}^{\prime \prime }}$.

Using the waveform data obtained in the analysis and experiment details in Section 3.2, using the surface reflection method (SRM), the storage elastic modulus $G^{{}^{\prime }}$ and the loss elastic modulus $G^{{}^{\prime \prime }}$ of the four rubber materials were obtained by Equation (22) and the result is shown in Figure 11.

**Figure 12:**
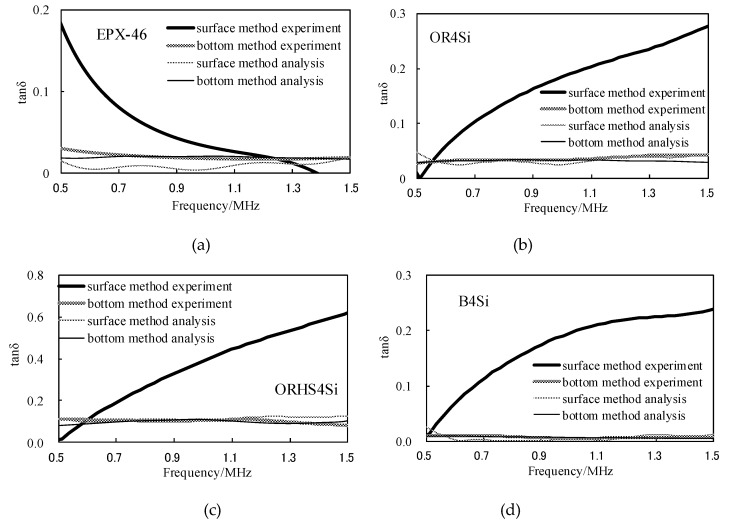
Comparison of different materials by SRM and BRM simulation and experimental viscoelastic evaluation results: (**a**) the tanδ of EPX-46; (**b**) the tanδ of OR4Si; (**c**) the tanδ of ORHS4Si; (**d**) the tanδ of B4Si.

The SRM does not require calculation of the attenuation coefficient. The results of the four methods (BRM experiment, BRM analysis, SRM experiment and SRM analysis) are compared through the loss tangent tanδ, as shown in Figure 12.

It can be seen from Figure 11 that the experimental measurement and the ultrasonic propagation analysis are compared. For the storage modulus $G^{{}^{\prime }}$, both the experimental and the simulation methods show good consistency, but due to the large difference between the results of experiments and simulations of loss elastic modulus $G^{{}^{\prime \prime }}$, the two results of the loss tangent tanδ have large errors. The reasons for this will be discussed in the next section. In Figure 12, the BRM results of the experiment and simulation have good consistency. The results of simulation by the SRM are slightly larger than the errors of the first two, but the same trend is observed. However, the results of the SRM are quite different from those of the first three.

### 4.2. Result Analysis and Optimization

**Figure 13:**
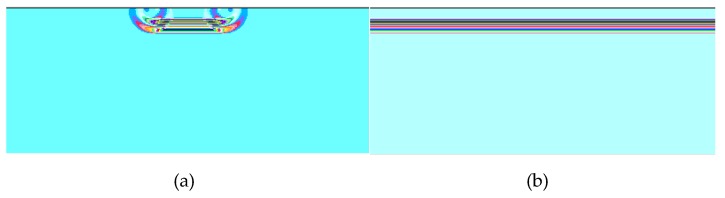
The difference in the wave motion pattern around the sensor corner, wave propagation with time in different incidence regions in the buffer material: (**a**) small-diameter sensor; (**b**) large-diameter sensor.

Theoretically, it can be predicted that the evaluation results of the BRM and the SRM are the same, but here, the cause of the above error is explored. In the model shown in Figure 6, a longitudinal wave is emitted from a sensor (with a diameter of 10 mm) to a buffer block. In this case, the longitudinal wave propagates within the diameter of the sensor, but shear stress is also generated around the sensor. That is, a mode conversion of a waveform is generated around the sensor, see Figure 13a, which shows an ultrasonic propagation simulation using a sensor with the same diameter as that used in the actual experiment. In the SRM process, the evaluation method is based on the surface reflected wave of the material to be evaluated. Unlike BRM, since the SRM does not use the interpolation error wave during ultrasonic propagation, it is sensitive to the waveform transformation around the sensor. Therefore, in the study, a uniform incidence model was used for the incidence of the longitudinal wave. The ultrasonic propagation condition, in this case, is as shown in Figure 13b. The propagation of waves in (a) and (b) is significantly different. It can be seen from Figure 13a that the plane wave propagates forward in the sensor diameter range, but the spherical wave is generated at both ends of the sensor. Based on the above research, it is expected that the spherical wave influences the receiving waveform; on the other hand, in Figure 13b, the plane wave propagates over the entire range of the medium. Therefore, ultrasonic wave propagation analysis was performed using the incident wave of Figure 13b, and the BRM and the SRM were compared.

### 4.3. Verification of optimization method

**Figure 14:**
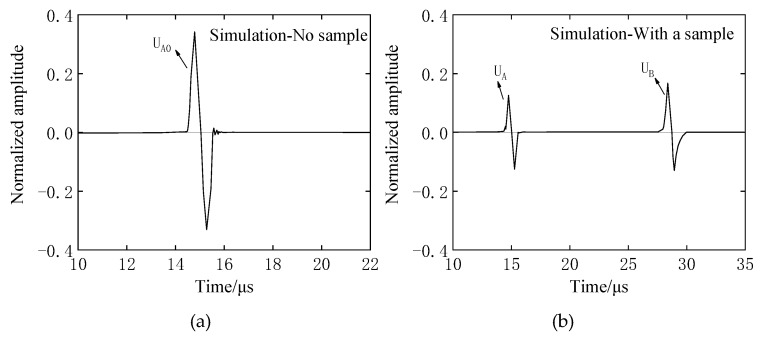
Other conditions are unchanged, and the simulation waveform of incidence and reflection by PZFlex analysis is optimized only by increasing the diameter of the sensor: (**a**) reflected waveform received without a sample; (**b**) reflected waveform received with a sample.

**Figure 15:**
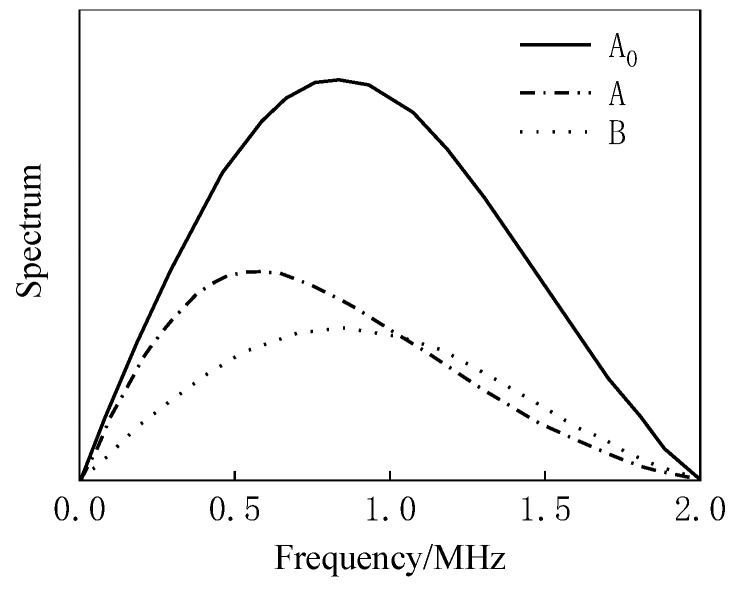
Frequency spectrum of waveform shown in Figure 14 after increasing the diameter of the sensor by PZFlex simulation.

In the case of uniform incidence in Figure 13b, the waveform of the bottom of the retarded material when there is no sample, the waveform of the bottom of the buffer material with a sample, and the waveform of the reflected wave on the bottom of the sample are shown in Figure 14a,b. The above ultrasonic wave propagation is analyzed, and the waveform of Figure 14 is subjected to Fourier transform analysis according to the above-described SRM and BRM principles, and the amplitude spectrums A_0_(*f*), A(*f*), and B(*f*) of the respective reflected waves are as shown in Figure 15. 

**Figure 16:**
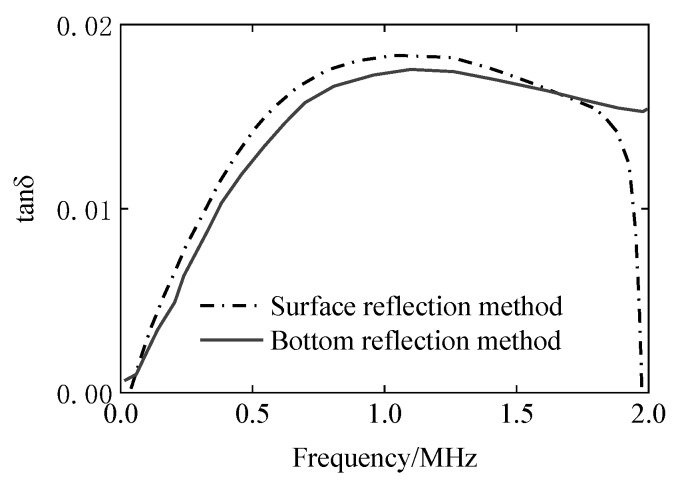
In the effective frequency range, the evaluation results of the viscoelasticity of the EPX-46 rubber material by SRM and BRM, that is, the tanδ value.

According to the results of Figure 15, the loss tangent values were calculated using SRM and BRM, and the viscoelasticity evaluation curve was obtained, as shown in Figure 16, the value of the SRM is slightly larger than the value of the BRM but is very consistent over the effective frequency range.

It can be confirmed from the research results that both the BRM and the SRM using the longitudinal wave can be applied to the viscoelastic evaluation of the material. However, in the actual measurement of the sensor, the mode conversion of the acoustic wave is avoided as much as possible by using a large-diameter sensor to ensure the effectiveness of the evaluation of the viscoelasticity of the material in the effective frequency range of the SRM and the BRM.

## 5. Conclusions

The ultrasonic longitudinal wave was transmitted into the viscoelastic rubber material, and the analysis based on the BRM and the SRM was performed using the reflection waveform from the material, and the characteristics of the viscoelasticity of the rubber material in the frequency response range were evaluated. The following results were obtained by a comparative study of the two methods’ experimental and analytical results.

(1) The ultrasonic method is used to evaluate the viscoelasticity of the material. It is not necessary to cut and destroy the material as non-destructive measurement can be realized. Among the available methods, the ultrasonic bottom reflection method cannot evaluate the viscoelasticity of the irregular bottom material, and the surface reflection method just makes up for the shortcoming. However, the evaluation of the viscoelasticity of the material by the ultrasonic method is easily affected by the coupling effect between the sensor and the material contact surface, such as the characteristics of the coupling agent, the flatness of the material contact surface, etc.

(2) In order to evaluate the viscoelastic properties of polymer materials, the BRM and the SRM using ultrasonic waves have been clarified from both experimental and analytical aspects. In particular, a surface reflection method using a longitudinal wave has been proposed, and for the incidence of the longitudinal wave plane wave, the error of the viscoelastic parameter obtained by the ultrasonic wave propagation analysis and the result of the BRM analysis is within 8%. It is clear that the longitudinal wave obtained by the SRM can be applied to the viscoelastic evaluation of polymer materials.

(3) In the process of exploring the viscoelasticity of rubber materials, the received signals are analyzed by spectrum analysis, and the approximate trends of storage modulus, loss modulus, attenuation and loss tangent in the effective frequency range are explored in the simulation and experimental calculation of the two methods.

(4) In the viscoelastic evaluation of the practical rubber material, ultrasonic propagation analysis and ultrasonic experimental measurements were simultaneously performed, and the reflected waveform obtained by the BRM and the SRM was evaluated to determine the viscoelasticity of the rubber. As a result, from the comparison of the ultrasonic wave propagation analysis and the experimental measurement, it was found that the four kinds of rubber materials obtained substantially uniform results by the BRM, but in the SRM, the difference between the analysis result and the experimental result was large. This error is mainly due to the fact that the mode conversion of the acoustic wave is generated around the ultrasonic sensor. Therefore, it is concluded that the practical performance of the SRM can be improved by increasing the diameter of the ultrasonic sensor.

## Figures and Tables

**Table 1:** Specification of material surface ultrasonic diagnosis analyzing system.

**Specification of the pulser/receiver**	
**Pulse configuration**	square burst pulse or chirp waves
Bandwidth	300 Hz~10 MHz
Low pass (MHz)	1.00, 2.00, 5.00
High pass (MHz)	0.01, 0.20, 1.00
Gain	0 to 80 dB
Output impedance	50 Ω
**Specification of the A/D transition**	
Sampling Clock	100 MHz
Analogue bandwidth	DC~50 MHz
Input impedance	50 Ω or 10 kΩ

**Table 2:** Physical property of specimen.

Sample Name	Filler	Hardness (HA) of Material	Glass Transition Temperature by DMA (°C)	Velocity (m/s)	Density *ρ*_2_ (kg/mm^3^)
EPX-46	Silica	41	−66	1463	0.98
OR4Si	Silica	45	−50	1546	1.077
ORHS4Si	Silica	48	−24	1723	1.106
B4Si	Silica	45	−105	1520	1.059

**Table 3:** The comparison of attenuation coefficient α by experiment and analysis at 1 MHz (BRM).

Sample	By Experiment	By Analysis	Difference Rate
**EPX-46**	0.377	0.402	6.31%
OR4Si	0.688	0.744	7.53%
ORHS4Si	2.036	2.228	8.62%
B4Si	0.102	0.114	10.68%

**Table 4:** The comparison of tanδ by experiment and analysis at 1 MHz (BRM).

Sample	By Experiment	By Analysis	Difference Rate
**EPX-46**	0.018	0.0198	9.2%
OR4Si	0.034	0.0357	4.6%
ORHS4Si	0.105	0.108	3.0%
B4Si	0.005	0.0056	10.7%
